# COVID-19 Intubation Safety: A Multidisciplinary, Rapid-Cycle Model of Improvement

**DOI:** 10.1177/1062860620949141

**Published:** 2020-08-18

**Authors:** Amy Tronnier, Collin F. Mulcahy, Ayal Pierce, Ivy Benjenk, Marian Sherman, Eric R. Heinz, Scott Honeychurch, Geoffrey Ho, Kendarius Talton, David Yamane

**Affiliations:** 1 The George Washington University, Washington, DC; 2 The George Washington University Hospital, Washington, DC

**Keywords:** COVID-19, intubation, safety, quality improvement, personal protective equipment

## Abstract

The COVID-19 pandemic has forced the health care industry to develop dynamic protocols to maximize provider safety as aerosolizing procedures, specifically intubation, increase the risk of contracting SARS-CoV-2. The authors sought to create a quality improvement framework to ensure safe practices for intubating providers, and describe a multidisciplinary model developed at an academic tertiary care facility centered on rapid-cycle improvements and real-time gap analysis to track adherence to COVID-19 intubation safety protocols. The model included an Intubation Safety Checklist, a standardized documentation template for intubations, obtaining real-time feedback, and weekly multidisciplinary team meetings to review data and implement improvements. This study captured 68 intubations in suspected COVID-19 patients and demonstrated high personal protective equipment compliance at the institution, but also identified areas for process improvement. Overall, the authors posit that an interdisciplinary workgroup and the integration of standardized processes can be used to enhance intubation safety among providers during the COVID-19 pandemic.

The COVID-19 pandemic has created numerous challenges within the sphere of clinical medicine and has forced health care providers (HCPs) and hospital administrators to develop novel safety protocols to ensure the well-being of HCPs and patients alike.^1^ With rapidly changing information and availability of personal protective equipment (PPE), safety protocols must be adaptable and constantly updated to incorporate new information made available by the global scientific community.^2^ Now more than ever, HCPs and administrators are relying on rapid-cycle improvement methodology to implement quality and safety initiatives in a timely manner.^3,4^ Moreover, significant evidence suggests that multidisciplinary cooperation and input from HCPs on the front lines are essential to creating meaningful and impactful protocols.^5^

HCPs performing intubations on patients with unknown, suspected, or confirmed COVID-19 are at the forefront of the current health care and public safety crisis. Multiple studies have demonstrated that aerosol-generating procedures place HCPs at high risk of contracting COVID-19, with endotracheal intubation believed to be particularly hazardous because of the provider’s close proximity to a patient’s airway throughout the procedure.^6-10^ Although the exact risk of transmission to those performing aerosolizing procedures in the midst of the current pandemic is not yet known, a systematic literature review and meta-analysis evaluating transmission risk during the 2003 SARS-CoV-1 (severe acute respiratory syndrome coronavirus 1) outbreak demonstrated that providers who performed intubations had >8 times the odds of developing SARS than those who did not (95% confidence interval 5.3, 14.4).^6,7,9,10^ Furthermore, it has been estimated that 8% of individuals infected with SARS-CoV-2 ultimately will require endotracheal intubation and mechanical ventilation.^6^ Although proper use of airborne precaution PPE is known to be effective in reducing the risk of nosocomial infection, it is also believed that additional implementation of engineering and administrative controls is essential to protecting those personnel performing intubations in patients with COVID-19.^6^

At the study institution, a tertiary care facility within an academic medical center in Washington, DC, the research team developed a multidisciplinary model centered on rapid-cycle improvements and real-time gap analysis to track adherence to intubation safety protocols established for providers treating patients with unknown, suspected, or confirmed COVID-19. The goals in implementing these quality measures were to improve intubation safety protocol adherence, understand deficiencies in PPE utilization, and provide expedited support to HCPs on the front lines.

## Methods

Institutional review board approval was obtained for the creation of a COVID-19 patient registry, capturing multiple clinical data points ranging from patient demographic information to ventilator settings. As part of this patient data registry effort, multiple third- and fourth-year medical student research assistants abstracted data from the electronic medical record (EMR) for COVID-19-positive patients admitted to the institution. Between March 24, 2020, and May 19, 2020, information on a total of 325 patients was entered into this registry.

A subset of this larger team was specifically responsible for tracking metrics pertaining to all intubations in this population, including monitoring adherence to established intubation safety protocols, and evaluating the ongoing availability of adequate PPE in the context of worldwide shortages.

### Research Setting

The quality improvement initiatives took place at The George Washington University Hospital, a 300-bed academic medical center with 700+ COVID-19 discharges to date. In the midst of the COVID-19 pandemic, while some institutions opted to create dedicated airway teams for COVID intubations,^11,12^ the study institution employed a different model, allowing multiple providers to perform these intubations with the guidance of a dedicated team of safety officers. Safety officers play a pivotal role in each of these procedures by ensuring proper donning and doffing of PPE, educating providers regarding any changes to hospital intubation protocols, and maintaining an overall environment of safety. In this hospital, intubations are generally performed by emergency department (ED) physicians (when the patient is in the ED), critical care physicians (when the patient is in the intensive care unit [ICU] or is rapidly decompensating outside of the ICU), and anesthesiologists (trauma activations, when the patient is in the operating room [OR], or any “difficult airway” in the hospital). With multiple teams performing intubations in different locations throughout the hospital, in order to prevent errors, the research team felt the strong need to develop a standardized approach and to employ cognitive aids to ensure that the processes were obvious and replicable.

### Quality Improvement Team Structure

The multidisciplinary team consists of 2 research coordinators, 6 medical students (third- and fourth-year students), 2 residents (otolaryngology, emergency medicine), a critical care advanced practice provider, and 3 attendings (critical care, emergency medicine, anesthesiology), with the support of 59 trained safety officers (registered nurses and physical therapists). Medical students responded directly to resident leads, who helped facilitate day-to-day operations. Attending leads developed the Intubation Safety Checklist (ISC) as well as the COVID-19 Intubation Note template (both described in the following section) and initiated the implementation of designed protocols in the ED, ICU, and ORs. Safety officers were present at all intubations. In addition to assisting with PPE and provider education, safety officers observed the intubation procedures and completed the ISCs. Research coordinators provided expertise in developing and maintaining the research database in addition to overall operational support and project leadership.

### Instruments

The primary instrument was the single-page COVID-19 ISC (supplementary Appendix 1, available with the article online), which was completed by the safety officers who were present for all suspected or confirmed COVID-19 intubations. This checklist monitors which articles of PPE were used during each intubation as well as the reasons for improper or inadequate PPE usage. It also includes a section for additional comments or concerns regarding intubation equipment, PPE, or personnel. In the ED, all intubations were considered suspect for COVID-19—including traumas, strokes, and other non-respiratory indications—unless there was already a negative test result from the current visit.

As a secondary instrument, the team developed a text messaging template (supplementary Appendix 2, available with the article online) for contacting providers via TigerConnect (TigerConnect Inc, Santa Monica, California), a secure messaging platform, following intubations. The goal was to contact providers within 24 hours of an intubation to collect feedback regarding any quality concerns or opportunities for process improvement from their perspective.

The third instrument used was a standardized note template (supplementary Appendix 3, available with the article online), which was disseminated to all potential intubating providers to incorporate in the EMR when documenting intubations in any known or suspected COVID-19 patients. The integration of this template allowed for more consistent documentation between providers and specialties while emphasizing certain parameters unique to intubating patients in this population.

### Quality Improvement Team Workflow

To capture feedback from intubations in real time, medical students created a rotating on-call schedule and monitored the EMR for intubations. When intubations occurred, documentation was reviewed, information gaps were identified, and medical students utilized TigerConnect to contact intubating providers to obtain any missing information. Simultaneously, providers were asked to include feedback regarding any safety concerns with regard to the procedure, or any perceived opportunities for systems-level improvement. When ISCs were completed at the time of intubation, safety officers had the option to store them in one of 5 predetermined secure locations in the hospital, with routine collection by one of the in-house team members. Alternatively, the individual completing the ISC could submit a photograph of the completed document electronically via a TigerConnect Role, established specifically for this purpose and monitored by the on-call team member. During the initial implementation phase of this project, resident and attending leads also were used to perform informal, in-house quality checks to monitor for compliance and promote consistent use of the ISC.

**Figure 1.**
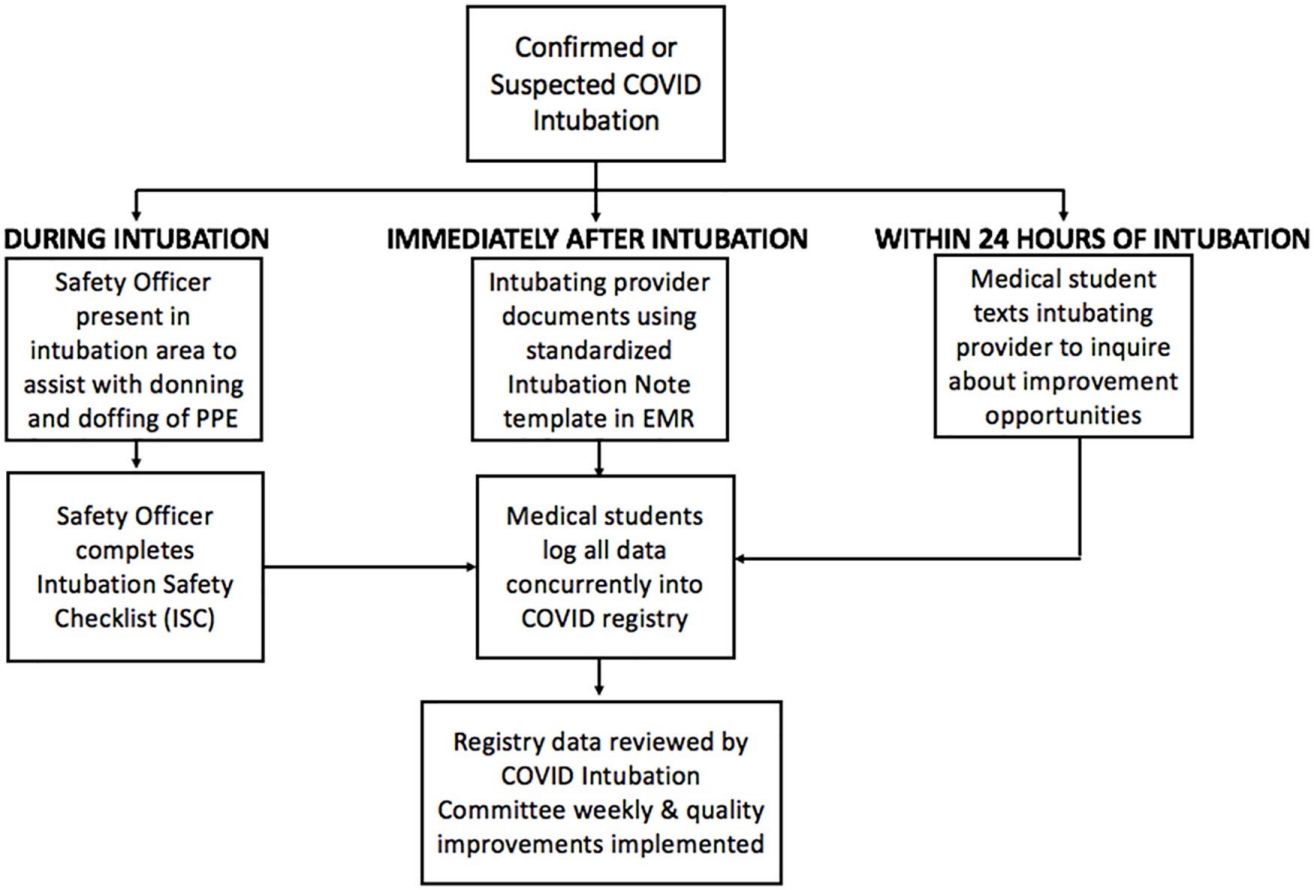
Intubation safety quality improvement team workflow. Abbreviations: EMR, electronic medical record; PPE, personal protective equipment.

Weekly team meetings with medical student research assistants were held by the resident leads via secure telecommunication platforms to discuss the progress of data abstraction and to problem-solve using a shared mental model. Additionally, weekly meetings among the attending leads, research coordinators, and resident leads were held to review data, identify information gaps, and implement quality improvements as needed. Figure 1 displays a diagram of the team workflow.

## Results

### Intubating Providers

Between April 15, 2020, and May 16, 2020, data were collected for 68 intubations performed in accordance with the COVID-19 intubation safety protocols (54 intubations in COVID-19-positive patients and 14 intubations in patients with unknown COVID-19 status at time of intubation who ultimately tested negative). These intubations were performed by 37 unique providers, yielding an average of 1.8 intubations per provider. Of these 68 intubations, 58.8% (n = 40) were performed urgently, 33.8% (n = 23) were performed emergently, and 7.4% (n = 5) were performed for the purpose of nonelective surgery. Supplementary details pertaining to these intubations can be seen in Table 1.

**Table 1.** Intubation Details.

**Provider specialty**	
Critical care (ICU)	41.2% (n = 28)
Anesthesiology	35.3% (n = 24)
Emergency medicine	23.5% (n = 16)
**Provider role**	
Attending	63.2% (n = 43)
Resident	30.9% (n = 21)
Fellow	5.9% (n = 4)
**Location of intubation**	
Intensive care unit (ICU)	58.8% (n = 40)
Emergency department	36.8% (n = 25)
Floor	2.9% (n = 2)
Operating room	1.5% (n = 1)

### Intubation Safety Checklist

The ISC was introduced in mid-April, and out of 54 COVID-19-positive intubations that took place at the institution between April 15 and May 16, 2020, the team received completed ISCs for 74.1% (n = 40). The team also received completed ISCs for 14 intubations that occurred in patients with unknown COVID-19 status, who ultimately tested negative. However, as these intubations were performed in accordance with the COVID-19 intubation safety protocols, these data were included in the QI meetings to learn from those experiences as well.

**Figure 2.**
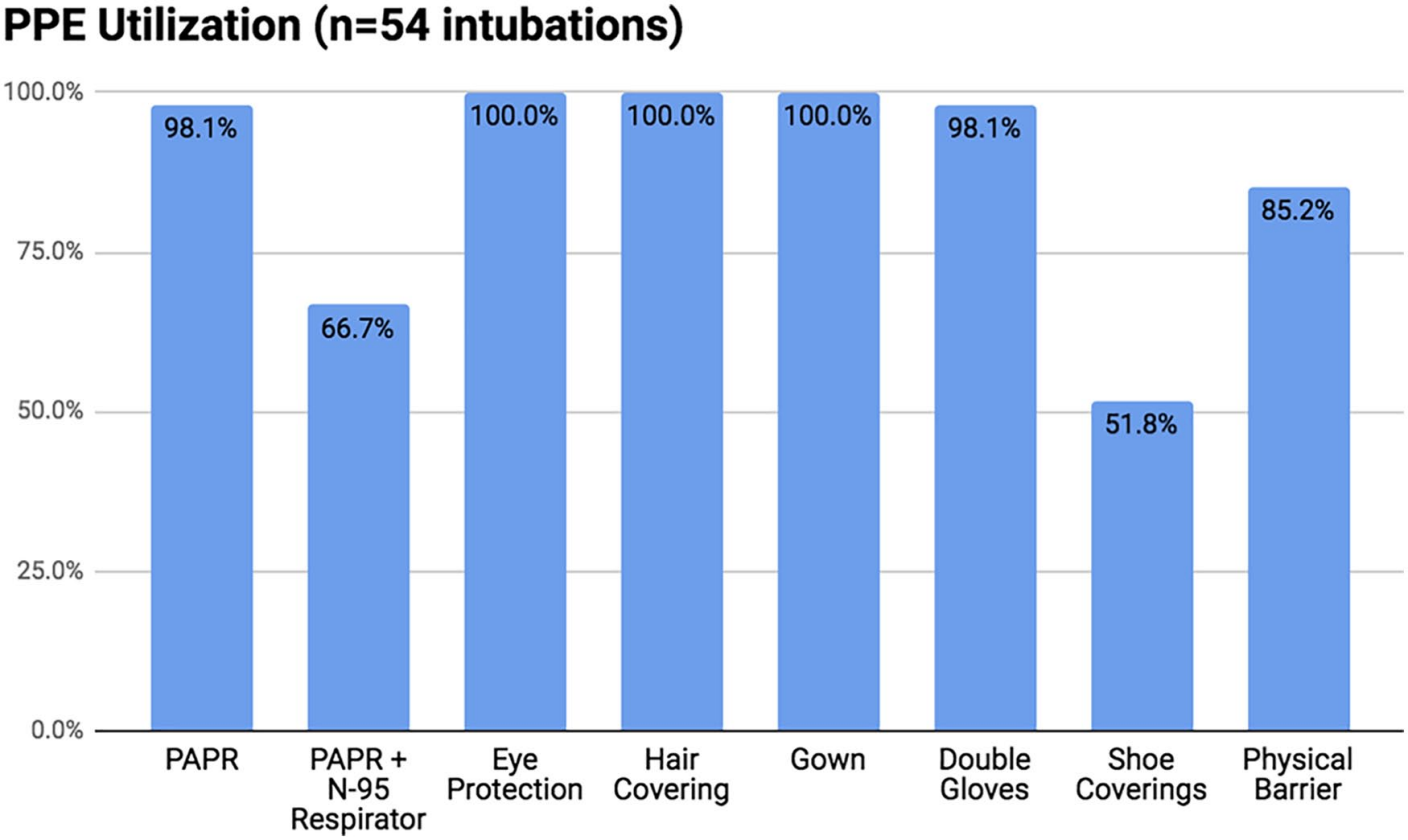
Personal protective equipment compliance data from ISCs for 54 intubations performed according to COVID-19 intubation safety protocols. Abbreviations: ISC, Intubation Safety Checklist; PAPR, powered air purifying device; PPE, personal protective equipment.

PPE compliance data are displayed in Figure 2. Based on the 54 completed ISCs, 98.1% (n = 53) of intubating providers wore a powered air purifying device (PAPR) and 66.7% (n = 36) also wore an N-95 respirator. The single provider who declined the use of a PAPR donned an N-95 respirator and chose to forgo using a PAPR due to the use of a sheet and hood covering the patient. All (n = 54) providers donned eye protection (either in the form of a face shield, goggles, or a PAPR) as well as a hair covering (in the form of a PAPR or bouffant), and a protective gown. Furthermore, > 98% wore 2 pairs of gloves, and in > 85% of the intubations, providers utilized an additional physical barrier between themselves and the patient (either a plastic sheet or intubation tent) to contain droplet spray. The article of PPE with the lowest reported compliance was shoe coverings (Figure 2). With regard to intubation technique, 64.7% (n = 44) of intubations were performed using a C-MAC video laryngoscope, 7.4% (n = 5) with a Glidescope, and 5.9% (n = 4) via direct laryngoscopy. In the remaining 22.1% (n = 15) of cases, the specific intubation technique was not documented.

### TigerConnect Follow-Up

Over the course of 4 weeks, medical students on the team contacted 21 intubating providers for follow-up and all but one replied, yielding a 95.2% response rate.

### Qualitative Feedback

**Figure 3.**
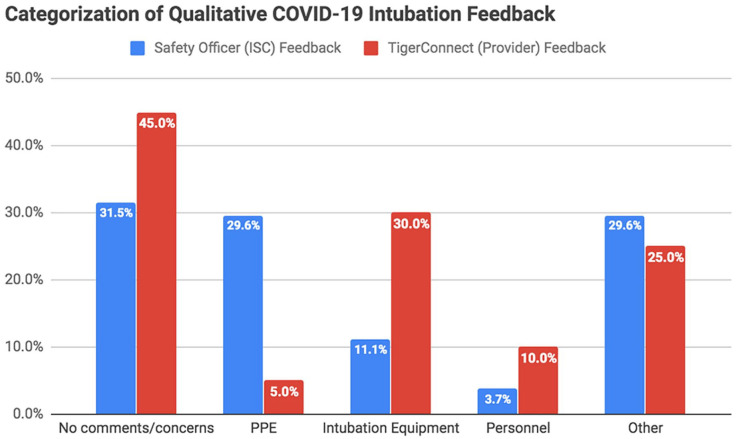
Categorization of qualitative feedback provided via ISC and TigerConnect communications. Abbreviations: ISC, Intubation Safety Checklist; PPE, personal protective equipment.

For all subjective feedback received via the ISCs or TigerConnect communications, comments were categorized as follows: no comments or concerns, PPE, intubation equipment, personnel, or other. The majority of the ISCs and TigerConnect communications yielded additional qualitative feedback (Figure 3). Although sometimes this feedback was positive and other times it identified opportunities for improvement, all feedback was reviewed for quality improvement purposes. The distribution of categorized feedback relative to the 2 feedback modalities (ISCs reflecting safety officer feedback and TigerConnect communication reflecting provider feedback) can be seen in Figure 3.

### COVID-19 Intubation Note Template

The standardized note template created for intubations of any known or suspected COVID-19 patients was used for documentation in 66.2% (n = 45) of the total cases. However, this template was used in 77.8% of the COVID-19-positive intubations, compared with only 21.4% utilization in the patients found to be negative for COVID-19 following intubation.

### Weekly Interdisciplinary Team Meetings

The team met weekly from April 19, 2020, to May 27, 2020, to review the qualitative and quantitative feedback collected. This process led to immediate actions aimed at rapid-cycle improvement. Some examples of comments and actions are listed in Table 2.

**Table 2.** Actions Taken in Response to Qualitative Feedback From Safety Officers and Intubating Providers.

Feedback received	Actions taken
Request to revisit workflow regarding recommendations concerning use of noninvasive ventilation, specifically the use of bag-valve-mask (BVM) ventilation during intubations	Following interdisciplinary team meetings, guidelines were clarified to reflect the following: ● When hypoxemia is critical, *judicious* use of BVM ventilation is acceptable ● As the use of BVM ventilation does change the safety profile of any individual in the room not donning a PAPR, an intubation tent should be used while bagging whenever possible ● If responding to a code on the floor for a patient without an established airway, BVM ventilation should not be used ● Routine BVM ventilation still strongly discouraged
Certain medications were not available in the rapid sequence intubation (RSI) bag	Worked with intensive care unit medical director to expand the number of medications routinely available in the RSI bags
Providers endorsed challenges to consistent and proper use of the added physical barriers while performing intubations	Provided additional education concerning use of intubation tents/plastic sheets over patients
In the emergency department (ED), many intubation supplies had been moved outside of procedure rooms to minimize potential contamination. Providers found this to be challenging as, on occasion, certain supplies were not gathered prior to donning PPE and entering the procedure room.	Through interdisciplinary meetings, anesthesia colleagues shared an adaptive tool they had developed for use when responding to Code Blue-19s (cardiac/respiratory arrest in a COVID-19 patient). What was termed a “*COVID Intubation-To-Go Bag*” was a preassembled, grab-and-go clear plastic bag containing the following intubation supplies: ETTs of different size (each with a stylette and a 10-cc syringe), a bougie, a disposable MAC3 laryngoscope blade, an LMA, and tape. See supplementary Appendix 4 (available with the article online) for a complete list of the supplies included. Upon learning of this challenge in the ED, anesthesia colleagues shared their prototype to-go bag with the ED charge nurse, who subsequently created a similar *ED Intubation Bag* containing essential airway supplies similar to those listed above. The prepared ED Intubation Bags were then stored by the entrance to the resuscitation bays for quick pick-up as needed.

Abbreviations: ETT, endotracheal tube; LMA, laryngeal mask airway; PAPR, powered air purifying device; PPE, personal protective equipment.

## Discussion

During a novel pandemic with a rapidly changing landscape, adaptable protocols for quality improvement in health care delivery have been crucial for success. At the study institution in Washington, DC, implementing a multidisciplinary model designed to monitor provider safety while promoting continued quality improvement in the face of a global pandemic has allowed for the implementation of rapid-cycle improvements in safety protocols for HCPs treating patients with unknown, presumed, or confirmed COVID-19.

Though an initial goal in implementing the ISC was to measure individual practice improvement and compliance with PPE, the team was encouraged to see that PPE compliance at the institution has been consistently high since the tracking efforts began. The team also recognizes that, in the face of worldwide shortages, this institution was fortunate to maintain an adequate supply of PPE so that monitoring compliance was possible. Additionally, with 37 unique providers having performed an average of only 1.8 intubations each in suspected or confirmed COVID-19 patients, there are not yet enough data points to meaningfully assess individual improvement.

Having a large number of providers capable of performing these intubations is advantageous in that increased personal risk does not fall disproportionately on a small subset of providers. However, given the relative infrequency with which each provider performed these intubations, the team believes this further supports the need to develop robust processes and infrastructure to support individuals performing these procedures under the novel and constantly evolving conditions. Because the institution has chosen to distribute the risk inherent to performing these procedures among a larger number of capable providers, it loses the benefit of establishing seasoned practice teams. However, the constant in all of the study intubations has been the presence of trained safety officers, who have been an invaluable asset. Providers have expressed feeling safer overall because of having safety officers present to continually educate them and to adopt the burden of ensuring the proper donning and doffing of adequate PPE. The multidisciplinary team received feedback, such as the following: “[Provider] was very appreciative of the safety officer and it was a controlled and well-planned intubation. . . Staff observed good donning and doffing of PPE,” “. . . safety officers were helpful with safe donning and doffing,” and “I had no quality concerns. Well-oiled machine, adequate PPE. It went well.”

With regard to PPE compliance, although use of shoe coverings while performing intubations was observed to be low (51.8%), the necessity of this article of PPE has not been demonstrated in the literature, nor is it currently listed as recommended PPE by the Centers for Disease Control and Prevention or the World Health Organization. In the interim, this recommendation was removed from the hospital policy, reflecting the ever-changing requirements. Safety officers were crucial in the ability to adapt to this change by communicating the new recommendation to providers.

It is worth noting that on the ISCs (completed by safety officers), 29.6% of the comments pertained to PPE usage (compared to only 5% of the comments made by the intubating providers). Examples of safety officer feedback included the following: “Gown doffed without observer in critical care bay,” “CPR sheet delayed going on patient,” and “[Provider] doffed outer gloves prior to exiting room. Once in doffing area, instructed to doff inner gloves with gown. PAPR then doffed by safety officer without provider touching it.” Conversely, in the TigerConnect feedback (completed by intubating providers), 30% of comments were focused on intubation equipment (compared to just 11.1% of the comments from safety officers). Examples of this feedback ranged from difficulties with equipment, such as the C-MAC and radios for communication, to requests for ready access to a broader spectrum of intubation tools and medications. In analyzing these different observations and perspectives concerning the same procedure, the team feels that this difference in distribution only further supports the benefit of multidisciplinary cooperation in enhancing overall safety and quality improvement.

As an example of the rapid-cycle improvement model working in real time, during the process of compiling ISCs, an internal systems error was identified, which the team was able to rapidly address. The team identified a subset of data points for patients (n = 14) with unknown COVID-19 status at the time of intubation who eventually tested negative. Because COVID-19-negative patients are not being included in the internal registry, the feedback concerning these intubations (all of which were performed under the presumption of COVID-19 positivity) was not initially made available for review in the weekly meetings. With identification of these missing data points, the data capture and analysis system was quickly revised by creating a separate de-identified database to log this feedback for routine review by the multidisciplinary team.

Furthermore, in addition to enhancing patient and provider safety, this multidisciplinary model provided an inherent opportunity to supplement medical student education for those whose clinical rotations were suspended as a result of the pandemic. The opportunity to collaborate on this project proved to be a unique way to engage medical students in the care of COVID-19 patients without compromising safety, while also providing valuable research experience and opportunities for mentorship.

### Buy-In

With any new initiative, especially one that adds additional administrative steps to a provider’s workflow, some degree of resistance or delay in adoption is to be expected. However, the fact that this initiative was centered on the safety of providers, willingness to participate was widespread.

Based on this experience, the team posits that when clinicians are facing a novel threat to their personal health as well as the health of their patients, if a protocol is designed to reduce their risk and elevate overall safety, buy-in is likely to be broad and nearly immediate. In the initial stages, failure to complete the ISC was more likely because of a lack of awareness than a willful decision not to complete it. Once the safety officers were integrated and began completing the checklists with providers, compliance improved rapidly. Having each of the pertinent departments represented on the QI team also facilitated direct intradepartmental communication to spread awareness and encourage participation. Furthermore, medical student research assistants were critical for communicating with safety officers as well as following up with providers to encourage participation, and also providing additional opportunity for clinicians to share their experiences with a team focused on quality improvement.

### Challenges

Because of unforeseen circumstances, during the initial data collection phase, all students contributing to this project lost remote access to the EMR for a period of approximately 2 weeks. As a result of restrictions on medical student presence in the hospital (to support both appropriate social distancing and PPE stewardship), with the loss of remote EMR access, students were unable to contact intubating providers for follow-up during this period of time. This delay elucidated a need within the institution to develop more robust processes concerning student EMR access in order to facilitate involvement in valuable research efforts without compromising student safety or patient privacy.

Additionally, in rare cases, providers would transition off of clinical service shortly following intubations, marking themselves unavailable to be reached via TigerConnect, which made it challenging to follow up within the goal time frame of 24 hours post intubation. One way that was discussed to overcome this would be to streamline a system for the on-call team members to be notified of intubations in real time, so that providers could be followed up with immediately.

### Future Directions

Possible future directions include surveying providers to gauge overall perceptions of intubation safety in the COVID-19 pandemic as well as the perceived utility of receiving follow-up communications post intubation. Additionally, the team plans to evaluate the need and feasibility of long-term integration of these quality improvement initiatives upon resumption of normal hospital operations.

## Conclusion

Given the magnitude and complexity of the current COVID-19 public health crisis, with providers continuing to be at the forefront in positions of markedly increased risk, the heightened need for adaptable safety protocols and demand for interprofessional collaboration is clear. Based on observations and practices implemented at this tertiary care facility in Washington, DC, the team proposes a novel model centered on multidisciplinary cooperation, rapid-cycle improvements, and real-time gap analysis to improve compliance with intubation safety protocols among HCPs during the COVID-19 pandemic.

## Supplemental Material

AJMQ949141_Supplementary_Appendices_CLN – Supplemental material for COVID-19 Intubation Safety: A Multidisciplinary, Rapid-Cycle Model of Improvement Click here for additional data file. Supplemental material, AJMQ949141_Supplementary_Appendices_CLN for COVID-19 Intubation Safety: A Multidisciplinary, Rapid-Cycle Model of Improvement by Amy Tronnier, Collin F. Mulcahy, Ayal Pierce, Ivy Benjenk, Marian Sherman, Eric R. Heinz, Scott Honeychurch, Geoffrey Ho, Kendarius Talton and David Yamane in American Journal of Medical Quality
