# Supplementary material for: COVID-19 Intubation Safety: A Multidisciplinary, Rapid-Cycle Model of Improvement
Source: Am J Med Qual. 2020 Aug 18;35(6):450–7. doi: 10.1177/1062860620949141 (PMC7672671; doi:10.1177/1062860620949141)
Supplement: AJMQ949141_Supplementary_Appendices_CLN – Supplemental material for COVID-19 Intubation Safety: A Multidisciplinary, Rapid-Cycle Model of Improvement [file AJMQ949141_Supplementary_Appendices_CLN.docx]

**Appendix**


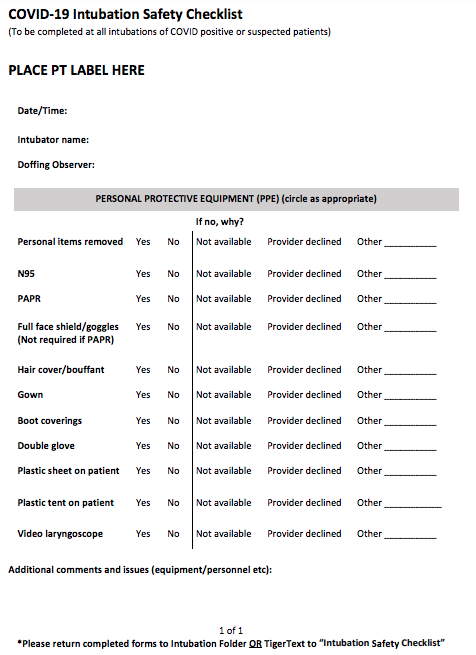


**Appendix 1:** Intubation Safety Checklist (ISC)

**
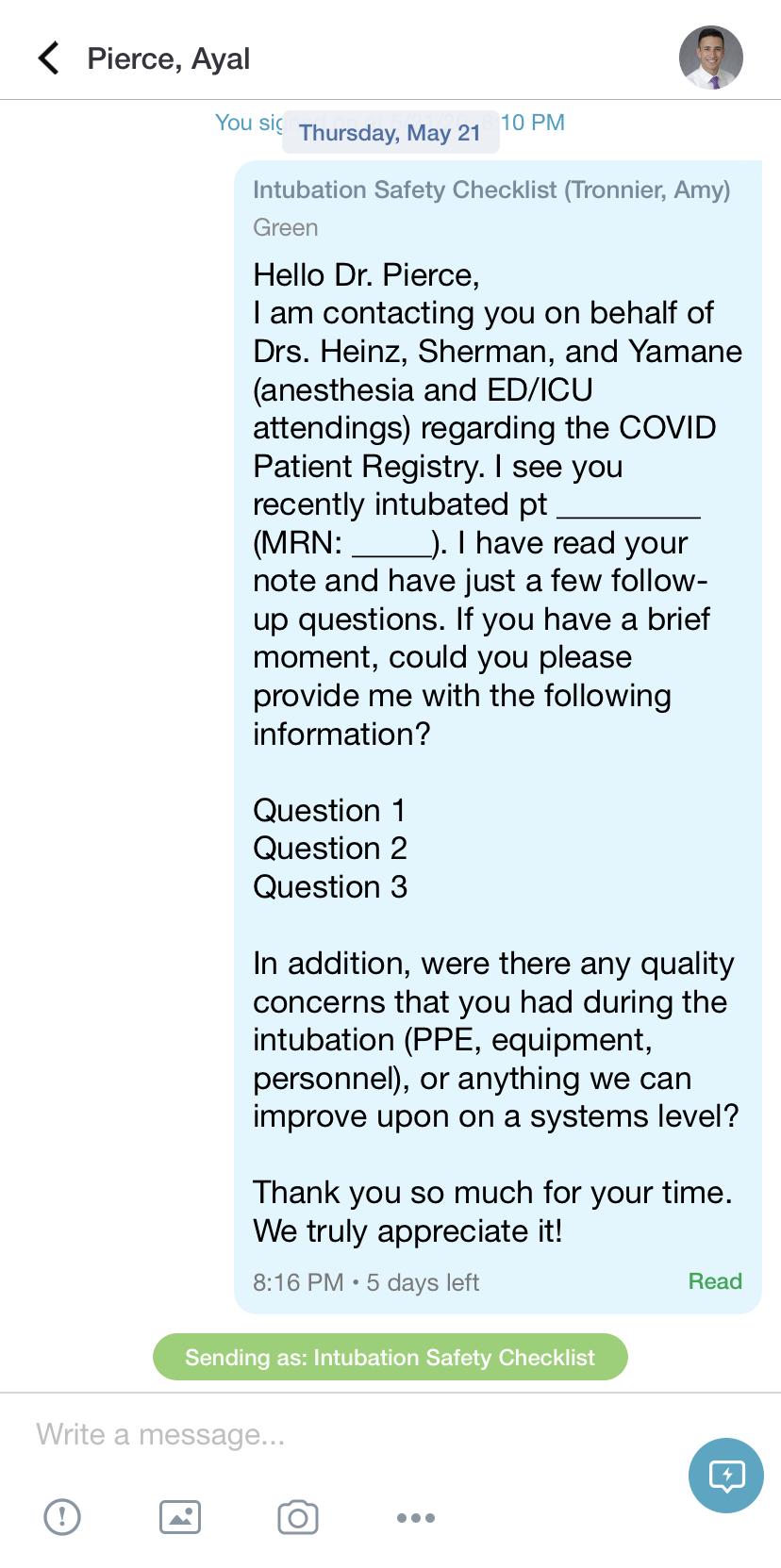
**

**Appendix 2:** TigerConnect^TM^ Template


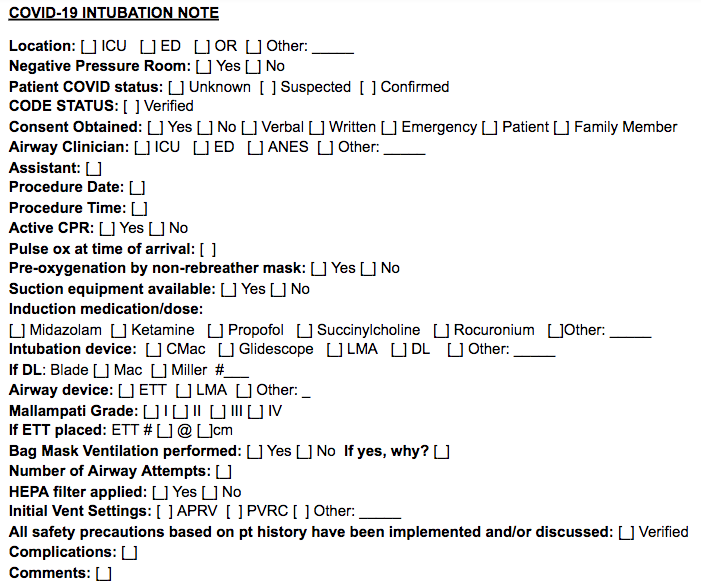


**Appendix 3:** COVID-19 Intubation Note Template

**COVID-19 Intubation To-Go-Bag**

- Two ETTs (7.5 and 8.0), each with stylette/syringe
- Eschmann/Bougie
- Oral airway
- Tongue depressor
- Tape
- Viral filter
- MAC3 blade (for backup only)
- LMA 4.5 (for backup only)

**ICU will provide**

- C-Mac, disposable blades, draped in plastic
- CO2 detector (colorimetric detector, CO2 sampling line)
- Ambu bag (for backup only)
- Induction/emergency medications
- Ventilator
- Respiratory Therapist with vent set-up (viral filter and ready circuit)

**Appendix 4:** Example of paper insert included in pre-assembled intubation kits developed by the anesthesiology department and later adapted for use in the emergency department
